# A Yeast RNA-Interference Pesticide Targeting the *Irx* Gene Functions as a Broad-Based Mosquito Larvicide and Adulticide

**DOI:** 10.3390/insects12110986

**Published:** 2021-11-02

**Authors:** Keshava Mysore, Longhua Sun, Limb K. Hapairai, Chien-Wei Wang, Jessica Igiede, Joseph B. Roethele, Nicholas D. Scheel, Max P. Scheel, Ping Li, Na Wei, David W. Severson, Molly Duman-Scheel

**Affiliations:** 1 Department of Medical and Molecular Genetics, Indiana University School of Medicine, Raclin-Carmichael Hall, 1234 Notre Dame Ave., South Bend, IN 46617, USA; kmysore@iu.edu (K.M.); Longhua.Sun.15@nd.edu (L.S.); limbh@pihoa.org (L.K.H.); jroethe@iu.edu (J.B.R.); mpscheel@iu.edu (M.P.S.); PLi@uams.edu (P.L.); Severson.1@nd.edu (D.W.S.); 2 Eck Institute for Global Health, The University of Notre Dame, Notre Dame, IN 46556, USA; cwang16@nd.edu (C.-W.W.); jigiede@nd.edu (J.I.); nscheel@wisc.edu (N.D.S.); nwei@nd.edu (N.W.); 3 Department of Civil and Environmental Engineering and Earth Sciences, The University of Notre Dame, Notre Dame, IN 46556, USA; 4 Department of Biological Sciences, The University of Notre Dame, Notre Dame, IN 46556, USA; 5 Department of Life Sciences, The University of the West Indies, St. Augustine, Trinidad, Trinidad and Tobago

**Keywords:** *Aedes albopictus*, *Aedes aegypti*, *Anopheles gambiae*, ATSB, *Culex quinquefasciatus*, insecticide, Iroquois, mosquito, RNAi, *Saccharomyces cerevisiae*, yeast

## Abstract

**Simple Summary:**

It is critical that we identify new methods of preventing mosquito-borne infectious diseases, which threaten millions of people worldwide. In this investigation, we describe characterization of a new insecticide that turns off the mosquito *Iroquois* (*Irx*) gene, which is required for mosquito survival. The pesticide is synthesized in yeast, which can be fed to adult mosquitoes in a sugar bait solution or to juvenile mosquitoes that eat the yeast when it is placed in water where mosquitoes breed. Although the yeast kills several different types of mosquitoes, it was not found to affect the survival of other types of arthropods that consumed the yeast. These results indicate that yeast insecticides could one day be used for environmentally friendly mosquito control and disease prevention.

**Abstract:**

Concerns for widespread insecticide resistance and the unintended impacts of insecticides on nontarget organisms have generated a pressing need for mosquito control innovations. A yeast RNAi-based insecticide that targets a conserved site in mosquito *Irx* family genes, but which has not yet been identified in the genomes of nontarget organisms, was developed and characterized. *Saccharomyces cerevisiae* constructed to express short hairpin RNA (shRNA) matching the target site induced significant *Aedes aegypti* larval death in both lab trials and outdoor semi-field evaluations. The yeast also induced high levels of mortality in adult females, which readily consumed yeast incorporated into an attractive targeted sugar bait (ATSB) during simulated field trials. A conserved requirement for *Irx* function as a regulator of proneural gene expression was observed in the mosquito brain, suggesting a possible mode of action. The larvicidal and adulticidal properties of the yeast were also verified in *Aedes albopictus, Anopheles gambiae*, and *Culex* *quinquefasciatus* mosquitoes, but the yeast larvicide was not toxic to other nontarget arthropods. These results indicate that further development and evaluation of this technology as an ecofriendly control intervention is warranted, and that ATSBs, an emerging mosquito control paradigm, could potentially be enriched through the use of yeast-based RNAi technology.

## 1. Introduction

Although insect control is the principal method of mosquito-borne disease prevention, insecticide resistance [1], combined with concerns for unintended negative impacts of insecticides on nontarget species [2], threatens ongoing international mosquito control efforts. The discovery of new classes of ecofriendly insecticides and new mosquito control techniques will help to ensure the future of successful mosquito control programs and arthropod-borne disease prevention [1,3]. The development of an adequate range of new insecticide classes is dependent upon accelerating the research and development of novel active ingredients and products for mosquito control [1]. To this end, RNA interference (RNAi)-based insecticides, a new class of insecticides for mosquito control, are presently being developed and evaluated [4,5]. RNAi is a conserved innate eukaryotic regulatory pathway that functions in response to double-stranded RNA (dsRNA), serving to protect organisms from exogenous pathogenic nucleic acids through the production of small interfering RNA (siRNA). siRNA silences expression of genes that are complementary in sequence through mRNA cleavage or translation inhibition [6]. Experimental applications for RNAi have permitted the functional characterization of genes in many different organisms, including mosquitoes [5,7]. RNAi technology could potentially be translated from the laboratory to the field, where recent efforts to extend this technology for agricultural [8] and disease vector insect control are gaining traction [5,7].

In mosquitoes, laboratory screens [9,10] have resulted in the discovery of small interfering RNAs (siRNAs) which target larval lethal genes, loci that are necessary for mosquito survival during the larval stages. Several of these larvicidal siRNA target genes are also required in adult mosquitoes and can, therefore, function as both larvicides and adulticides [11,12]. A subset of the siRNAs match target sites that are conserved in *Aedes* (dengue, chikungunya, yellow fever, and Zika vector), *Anopheles* (malaria vector), and *Culex* (lymphatic filariasis and West Nile vector) mosquito species, but which have not yet been identified in other genome sequences, including humans, as well as pollinators such as honey bees [12,13,14]. Ongoing characterization of these interfering RNAs and larval/adult lethal loci has supported the hypothesis that interfering RNA pesticides (IRPs) will kill several different species of mosquitoes at multiple stages of the mosquito life cycle yet pose little threat to nontarget species. The present investigation further examines this hypothesis through characterization of a putative larvicidal and adulticidal IRP with a target site that is conserved in mosquito *Iroquois* (*Irx*) family genes and which lacks an identical known target site in the genomes of nontarget organisms.

*Irx* family genes, which encode Iroquois-class homeodomain-containing proteins, are members of the TALE subfamily and components of the *Iroquois* gene complex, which is well conserved from insects through vertebrate organisms [15]. The *Irx* complex functions to regulate transcription, controlling territory and cell fate specification decisions, pattern formation, and cell-sorting behavior [16]. *Irx* family genes were initially discovered in *D. melanogaster* (reviewed by [15]), in which the function of the *Irx* complex is required for viability [15,16], supporting the hypothesis that *Irx* silencing in mosquitoes could result in death. Here, this hypothesis was evaluated during both the larval and adult stages in several species of disease vector mosquitoes through oral RNAi experiments, which were conducted using a yeast strain that expresses short hairpin (shRNA) that silences mosquito *Irx* genes.

Laboratory evaluation of several different interfering RNA delivery mechanisms resulted in the identification of baker’s yeast (*Saccharomyces cerevisiae*) as a promising method for oral transfer of interfering RNA to mosquitoes. *S. cerevisiae* is an excellent system for producing interfering RNA [4], and yeast is a potent odorant attractant for both gravid adult mosquitoes, which are lured to lay eggs in yeast-treated containers [17], as well as mosquito larvae, which readily consume larvicidal yeast upon hatching [9]. Moreover, the selection of *S. cerevisiae*, a model organism that is amenable to genetic manipulation, has facilitated generation of multiple yeast interfering RNA larvicide strains, each one targeting a different gene required for mosquito survival, resulting in the creation of an arsenal of yeast IRPs to combat pesticide resistance [4,5]. Importantly, the insecticidal properties of the RNA are preserved when the yeast is heat-inactivated, a key finding which would potentially allow the use of dead microbials, rather than live genetically modified organisms, to control mosquitoes in the field [9]. Production of interfering RNA through yeast culturing is expected to significantly reduce the costs of this intervention at scale, and fermentation is easily expanded from small laboratory-sized shake cultures to industrial scale [4]. *S. cerevisiae,* which is not toxic to humans, is utilized worldwide for beverage and food production and has been cultivated globally for thousands of years, suggesting that this yeast technology is readily adaptable for use in resource-limited regions of the world. Yeast can also be packaged and shipped without difficulty, which can enable global distribution of yeast pesticides [4]. For these reasons, recent efforts have focused on the potential for translating use of RNAi-based yeast larvicides for mosquito control from the lab to the field [5].

In addition to characterizing a new broad-based mosquito larvicide targeting *Irx* genes, here, we explore the potential use of yeast as the insecticidal component of attractive targeted sugar baits (ATSBs) for control of multiple adult mosquito species. ATSBs, a new mosquito control paradigm, take advantage of the innate sugar feeding behavior of female and male mosquitoes that are drawn to consume a sugar source containing an insecticide. ATSBs, which are supplied through bait stations or as sprays that can be used to treat foliage or bed nets, can be used both inside and outdoors [18], and field trials indicate that this cost-effective strategy will significantly advance mosquito control efforts [19,20,21,22]. ATSBs containing a variety of different broad-based insecticides, e.g., boric acid, dinotefuran, eugenol, and garlic oil, are being used for successful targeting of *Aedes* mosquitoes [23,24,25,26,27,28,29]. Likewise, various *Culex* species have been effectively controlled with ATSBs that deliver insecticides such as dinotefuran, boric acid, eugenol, encapsulated garlic oil, and Spinosad [24,30,31,32,33], and ATSBs targeting *Anopheles* mosquitoes are being developed as a mechanism for addressing residual malaria transmission [20,22,34]. Although ATSBs are a highly promising technology that will greatly facilitate targeted delivery of a number of different insecticides, insecticide resistance nevertheless remains to be a concern. Notwithstanding the addition of protective barriers to bait stations [35] and attempts to limit ATSB treatments to nonflowering plants, it is hard to eradicate all risks to nontargets, such as pollinator insects, while using currently available ATSB pesticide formulations, which do not uniquely target mosquitoes [18]. Here, we investigate the potential for using yeast IRPs as a novel class of insecticides that could significantly enhance ATSB technology. In this study, we aimed to develop and characterize a yeast interfering RNA strain that targets the mosquito *Irx* gene. We evaluated the yeast as a mosquito larvicide, and then developed and tested a yeast RNAi-ATSB delivery system for targeting adult insects of the three major genera of disease vector mosquitoes. We also examined a mode of action for the yeast in the mosquito nervous system and assessed the impact of yeast treatments on nontarget arthropods.

## 2. Materials and Methods

### 2.1. Mosquito Stains and Rearing

The following strains of mosquitoes were used in this investigation: *A. albopictus* Gainesville (BEI Resources, NIAID, NIH: MRA-804, donated by Sandra A. Allan), *A*. *aegypti* Liverpool-IB12 (LVP-IB12), *A. gambiae* G3 (BEI Resources, NIAID, NIH: Eggs, MRA-112, furnished by Mark Benedict), and *C. quinquefasciatus* JHB (supplied by the CDC to be distributed by BEI Resources, NIAID, NIH: Eggs, NR-43025). The strains were cultured as previously described [36] in an insectary maintained under the following conditions: 26.5 °C, ~80% relative humidity, and with a 12 h dark/12 h light cycle which incorporated a 1 h crepuscular period at the beginning and end of each cycle. An artificial membrane (purchased from Hemotek Limited, Blackburn, UK) was used for delivery of sheep blood purchased from HemoStat Laboratories, Dixon, CA, USA.

### 2.2. Discovery of siRNA #447

siRNA #447, which contains a target sequence identified in the *Irx* genes of multiple species of mosquitoes (see details in Table S1), was initially screened in larval soaking [9,10,37] and adult microinjection assays [11,12] that were completed in *A. aegypti* as previously discussed. Soaking experiments, which were performed in two replicate trials, were completed using 20 first instar (L1) larvae which were soaked in 20 uL of 0.5 μg/μL of siRNA #447 or control siRNA (custom synthesized by Integrated DNA Technologies, Coralville, Iowa) for 4 h. Following soaking treatments, the larvae were reared and evaluated as detailed in the World Health Organization (WHO) larvicide testing guidelines [38], and data were evaluated with the Fisher’s exact test. siRNA sequences were as follows: siRNA #447: 5′–AAAAAACCAAACGGGCAGCGACUGU–3′, control: 5′–GAAGAGCACUGAUAGAUGUUAGCGU–3′ [39]. For assessing the adulticidal activity as previously described [11,12], 20 3 day old non-blood fed adult females per treatment were anesthetized using carbon dioxide and microinjected in the thoracic region with 250 nL of 9 μg/μL *Irx*.447 or control siRNA (Integrated DNA Technologies, Coralville, IA, USA), after which time the mosquitoes were put in a cage for recovery. Adult mortality was subsequently evaluated every day for the next 6 days.

### 2.3. siRNA-ATSB Trials in Adults

ATSB trials with siRNA were completed as previously described [11,12] using 64 μL of 2.5 μg/μL siRNA in 10% sucrose solution containing 4.5% of red tracer dye (McCormick) which was dispensed from a cotton wick placed in a cut 0.2 mL microcentrifuge tube hung in a 3.75 L mosquito cage (Berry Global, Evansville, IN) located in the insectary. Three replicate trials were performed using 25 4–5 day old non-blood-fed adult females which were sugar-starved for 48 h prior to sugar bait feedings that were initiated at dawn and conducted for 4 h. Feeding was verified on the basis of the presence of red dye in the abdomen. Females that had sugar fed were evaluated daily for 6 days, after which time feeding rates were statistically evaluated using the G-test of independence, and the log-rank test was used for comparison of survival rates among treatments.

### 2.4. Yeast Larvicide Strain Generation and Culturing

Custom DNA oligonucleotides encoding an shRNA expression cassette that corresponds to *Irx*.447 target site 5′–AAACCAAACGGGCAGCGACTG–3′ were purchased from Invitrogen Life Technologies (Carlsbad, CA) and used in the generation of transformants bearing shRNA expression cassettes stably integrated at both the *ura3* and *trp1* sites of the *S. cerevisiae CEN.PK* strain [40] as previously described [9]. This yeast strain, which is referred to as *Irx*.447 yeast, as well as a similar control shRNA expression strain constructed in a previous study [9], was cultured for preparation of 50 mg tablets of dried inactivated yeast larvicide as described [41]. For ATSB trials, yeast was cultured in a similar manner, except that it was lyophilized in a Labconco FreeZone 6 L Console Freeze Dryer after culturing and pelleting, and then used for ATSB production and trialing as detailed below.

### 2.5. Larvicide Trials

Evaluation of larvicides was performed according to the WHO testing guidelines [3] as described [41]. Each of 18 replicate container trials was performed using 20 first-instar larvae (*n* = 360 larvae total per treatment) that were reared in 50 mL volumes of distilled water placed in 500 mL sized containers, along with a single 50 mg yeast tablet (either *Irx*.447 or control) that was available at the onset of each trial. Larvae were appraised throughout the trial period. At the end of the trial, larval mortality percentages were transformed using arcsine transformation as recommended prior to analyzing data with the Student’s *t*-test. Dose–response curves were generated and analyzed as previously described [9] using varying amounts of insecticidal and control interfering RNA yeast to prepare tablets with different doses of larvicide that were evaluated in larvae.

Semi-field evaluations of larvicides were performed according to the WHO larvicide testing guidelines [38] on an outdoor rooftop laboratory during July and August 2019 in Notre Dame, IN as previously described [13,14]. During the trial period, the average relative humidity was 75% ± 15%, and outdoor temperatures ranged from 9–35 °C, with a mean daytime temperature of 24 ± 5 °C and a mean nighttime temperature of 19 ± 4 °C. Each of 19 replicate container trials was performed using 20 LVP-IB12 strain *A. aegypti* mosquitoes that were placed in 7.5 L buckets (diameter = 23 cm, height = 25 cm) with 3.5 L of water and a 50 mg larvicidal or control yeast tablet. After the trials, the larval mortality rates in larvicide- or control-treated containers were transformed with arcsine transformation, and data were evaluated using a Student’s *t*-test.

### 2.6. Yeast ATSB Assays

For performance of *A. aegypti* simulated field trials, which were conducted in the insectary, yeast ATSB was prepared by homogenizing 40 mg of lyophilized yeast (*Irx*.447 or control) which contained 0.1% benzoic acid preservative with a 5% sucrose solution containing 0.05% Phytagel brand gellan gum (Sigma Aldrich, St. Louis, MO, USA; used to hinder ATSB desiccation) that was marked through the addition of 4.5 μL of red dye (McCormick’s) to a total volume of 100 μL that was placed in a 1.5 mL microfuge tube. For experiments conducted with *A. gambiae*, which are smaller than *Aedes* mosquitoes, the amount of yeast was halved to 20 mg per 100 μL of ATSB. Feeders were prepared by scoring the bottom of the 1.5 mL microfuge tube, which was then capped and perforated prior to hanging the yeast wick feeder at the top of the experimental cage, which was placed in the insectary. A total of 25 non-blood-fed 5–6 day old adult females that were sugar-starved for 2 days were placed in 3.75 L insect cages (Berry Global, Evansville, IN, USA), where they were permitted to eat from the two feeders for 4 h. Negative controls included ATSB with control yeast or ATSB with no yeast. After completing three replicate trials, mosquito feeding rates were assessed using the G-test of independence, and survival rates were evaluated using ANOVA. *Irx*.447 dose–response curves were generated and evaluated as described for the yeast larvicides in Section 2.5.

### 2.7. Whole-Mount In Situ Hybridization

The Patel [42] protocol was used to prepare riboprobes used to verify silencing of *Irx* genes (Table S1). Probes corresponding to the *A. aegypti POU domain protein 2* (*pdm2, AAEL017445*) and *A. gambiae pdm2* (*AGAP009500*) genes were also synthesized and used to assess the *Irx*.447 mode of action. The probes were used for detection of *Irx* and *pdm2* transcripts in adult female brains through in situ hybridization experiments, which were conducted as previously described [43]. Three biological replicate experiments were performed, and results were viewed and imaged with a Zeiss Axioimager (Carl Zeiss Microscopy, LLC, Thornwood, NY, USA) equipped with a Spot Flex imager (Diagnostic Instruments, Inc. Sterling Heights, MI, USA). Images were processed using FIJI ImageJ software [44], which was used to assess mean gray value (average signal intensity over a specified area) data, allowing for quantification of digoxigenin-labeled transcripts and data analysis using the Student’s *t*-test as described [45].

### 2.8. Evaluation of Nontarget Species

Yeast IRP toxicity was evaluated as described [11,12] in *Daphnia magna, Drosophila melanogaster,* and *Tribolium castaneum.* Toxicity assays were performed in *Hippodamia convergens* and *Oncopeltus fasciatus* according to the procedures described below.

*O. fasciatus* adults were acquired from Carolina Biologicals (Burlington, NC, USA) and cultured as described by the provider. For toxicity tests, which were performed in duplicate, a slurry of 200 μL of 10% sucrose combined with red marker dye and 50 mg of either *Irx*.447 or control interfering RNA yeast was provided to 20 adults (the total amount of yeast consumed per insect was the same as that used in mosquito assays). A 0.5 mL tube with a wick was suspended from the cage (which was maintained at room temperature, 21 °C) for delivery of the slurry to the insects throughout a six day trial period. Feeding was verified through observation of feeding bouts, as well as through observation of red marker dye in the insect feces. Survival data were analyzed using Fisher’s exact text.

*H. convergens* adults (Carolina Biologicals, Burlington, NC, USA) were reared in cages maintained at room temperature (21 °C) as directed by the supplier. Toxicity assays were conducted as described above for *O. fasciatus,* but were completed using 10 insects that fed on yeast ATSB which had been provided in a small dish throughout the trial period.

## 3. Results and Discussion

### 3.1. Silencing Irx Kills A. aegypti Mosquitoes

*Irx*.447 siRNA matches a conserved target sequence in *Irx* family genes of multiple mosquito species (Table S1) [46]. An identical sequence was not identified in the sequenced genomes [47] of other organisms (Table S1). The potential for *Irx*.447 siRNA to function as an insecticide was first evaluated in *A. aegypti,* in which siRNA soaking treatments resulted in significant larval mortality (Table 1). Significant mortality was also seen in *A. aegypti* adult females that were microinjected with *Irx*.447 siRNA in the adult thorax (Table 1). Given the results of these microinjection experiments, the potential for delivery of *Irx*.447 IRPs through ATSBs was then evaluated in a simulated field study conducted in the insectary in which a previously described sugar bait delivery system [11,12] was used for oral transfer of the *Irx*.447 siRNAs to adult females. Feeding rates of *A. aegypti* females following 4 h of exposure to *Irx*.447 sugar bait or sugar bait mixed with control siRNA (which has no known target in mosquitoes [39]) are shown in Table S2 and were similar to those observed in comparable trials with other siRNA adulticides [11,12]. *A. aegypti* feeding rates were not significantly different among the treatments (*p* > 0.05). Although no significant mortality was observed in adult female mosquitoes that consumed sugar bait alone or containing control siRNAs, significant mortality, 75% ± 3%, was noted in adult female mosquitoes that consumed *Irx*.447 siRNA ATSB (Table 1). These results in *A. aegypti* indicated that *Irx*.447 siRNA is an insecticide that has both larvicidal and adulticidal properties.

### 3.2. Delivery of the Yeast Pesticide as an ATSB

**Figure 1:**
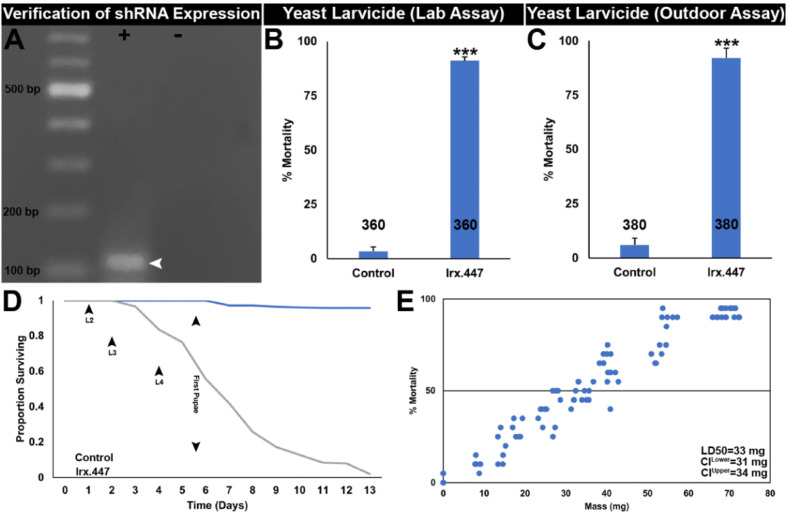
*Irx*.447 yeast consumption results in *A. aegypti* larval death. (**A**) A ~100 bp band amplified with primers corresponding to *Irx*.447 is seen in the lane marked by + in an agarose gel stained with ethidium bromide; cDNA prepared from *Irx*.447 yeast total RNA was the template in these reactions. No amplicon was detected in a negative control PCR reaction which lacked a cDNA template (marked by a minus symbol). A representative gel from two comparable biological replicate assays is displayed; irrelevant lanes were cropped from the image. (**B**) Consuming Rbfox1.457 yeast throughout larval development caused significant larval death in laboratory insectary trials, as well as in (**C**) semi-field outdoor trials conducted on *A. aegypti* larvae placed in 7.5 L buckets containing 3.5 L water. In (**B**,**C**), data combined from multiple replicate trials (each with 20 larvae) are displayed as mean percentages of larval mortality. (**D**) An *A. aegypti* larval survival curve corresponding to the data in panel (**B**) is shown. (**E**) A dose–response curve illustrates a positive correlation between *A. aegypti* larval mortality and the amount of *Irx*.447 yeast contained in larvicide tablets in which control and larvicidal yeast were mixed in varying proportions; each data point in (**E**) corresponds to the percentage mortality found in a single-container assay with 20 larvae; LD_50_ values are indicated. In (**B**,**C**), *n* numbers are shown under each bar in the graphs, and error bars denote SEMs; *** *p* < 0.001 (Student’s *t*-test).

The larvicidal and adulticidal activity of *Irx*.447 siRNA (Table 1) suggests that this insecticide could potentially be used to control mosquitoes. However, the present high costs of siRNA synthesis could impede broad deployment *Irx*.447 siRNA insecticides [5]. As noted above, this has been addressed through use of an *S. cerevisiae* shRNA expression system, which has been developed for potential use in larval control programs [4], but could potentially be deployed for use as an ATSB. In this investigation, development and evaluation of RNAi yeast targeting *Irx* facilitated examination of the hypothesis that yeast IRPs can be utilized as both larvicides and adulticides. *S. cerevisiae*, in which shRNA corresponding to the *Irx*.447 siRNA (hereafter referred to as *Irx*.447 yeast) was expressed through the stable integration of two *Irx*.447 shRNA expression cassettes, was used. PCR amplification of cDNA corresponding to the 3′ end of the hairpin and the terminator sequence resulted in a band of the expected ~100 bp size (Figure 1A).

Prior to evaluation of the putative adulticidal activity of IRP.447 yeast, the insecticidal activity of the *Irx*.447 yeast strain was verified in larvae, in which it induced 91% ± 2% larval mortality in indoor trials (Figure 1B; *p* < 0.001 vs. control interfering RNA larvicide treatment) and 92% ± 5% larval mortality in outdoor semi-field trials (Figure 1C; *p* < 0.001 vs. control interfering RNA larvicide treatment). Although control-treated larvae survived through adulthood, most *Irx*.447 larvae died during the fourth larval instar (a survival curve is shown in in Figure 1D). Higher dosage of *Irx*.447 increased the rates of larval mortality (Figure 1E), with the LD_50_ determined to be 33 mg. These results, combined with previous studies [9,11,12,13,14], demonstrate that yeast IRPs function as potent larvicides and add *Irx*.447 to the growing arsenal of yeast IRPs.

**Figure 2:**
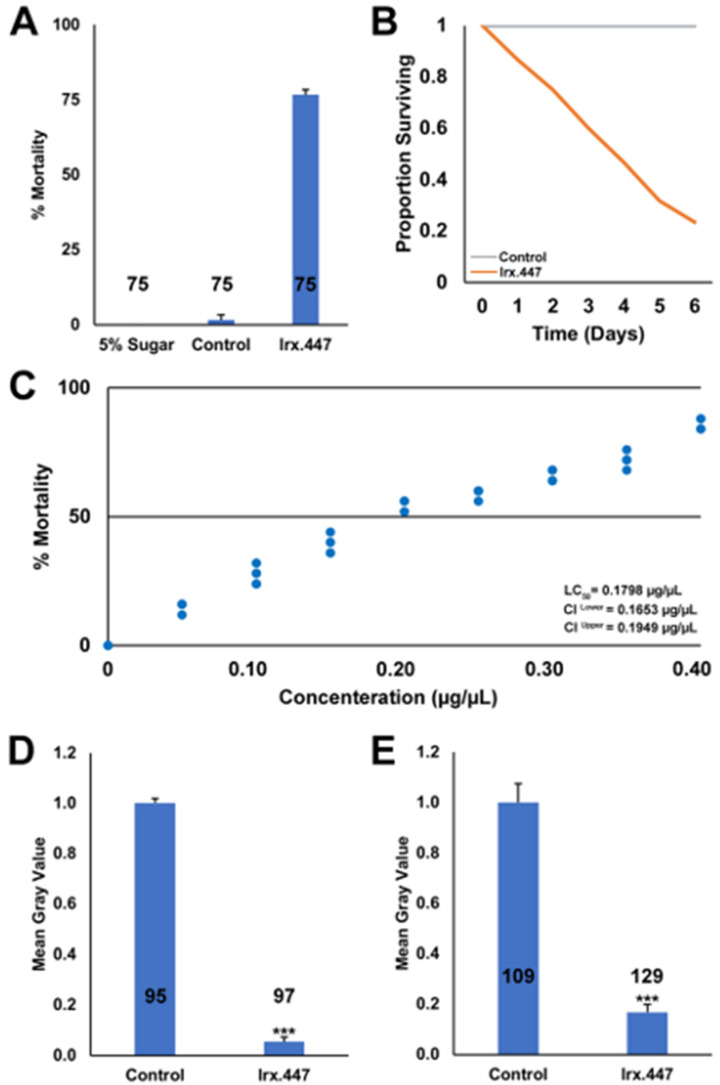
High *A. aegypti* mortality rates result from consumption of yeast RNAi-based ATSBs targeting *Irx*. (**A**). Significant mortality is observed following consumption of heat-inactivated *Irx*.447 yeast delivered to *A. aegypti* adult females as an ATSB. (**B**). Survival curves for adult females that consumed *Irx*.447 or control yeast sugar bait (in panel **A**) are displayed. (**C**). A dose—response curve illustrates the concentration of *Irx*.447 yeast provided in the ATSB vs. the percentage mortality of *A. aegypti* adult females; each point corresponds to an ATSB trial conducted on 25 adult females. LD_50_ values are indicated. *Irx*.447 ATSB consumption by *A. aegypti* adult females resulted in significantly reduced levels of *Irx* (**D**) and *pdm2* (**E**) transcripts in the brain, as evidenced by mean gray value analyses. Throughout this figure: *n* numbers are displayed under each bar in the graphs, and error bars denote SEMs; *** *p* < 0.001 (Student’s *t*-test).

Based on the successful verification of *Irx*.447 insecticidal activity in larvicide trials (Figure 1), the yeast was used to develop an *Irx*.447 yeast ATSB, which was evaluated in adult mosquitoes in simulated field trials conducted in the insectary. Yeast ATSB feeding rates were nearly doubled with respect to siRNA-ATSBs (Table S2), with *Irx*.447 yeast consumption verified in 100% of adult female *A. aegypti* mosquitoes (Table S2). This resulted in 77% ± 2% mortality (Figure 2A, *p* < 0.001 compared to control yeast in 5% sugar bait), which is comparable to the mortality rates observed in *Irx*.447 siRNA ATSB trials (Table 1). Although the mortality rates induced by *Irx*.447 yeast ATSBs are slightly less than what has been observed for siRNA-ATSBs targeting *dop1* and *Shaker* [11,12], the increased feeding rates associated with the yeast ATSBs, as well as the anticipated decreased production costs of yeast, make it an appealing delivery system. *Irx*.447 yeast ATSB treatments killed a majority of *A. aegypti* adult female mosquitoes within 6 days following ATSB consumption (Figure 2B). The percentage of *A. aegypti* female mortality correlated with the concentration of *Irx*.447 yeast in the ATSB (Figure 2C), with the LD_50_ value determined to be 0.18 µg/µL ATSB.

The mode of action for *Irx*.447 IRPs was next examined. In *D. melanogaster,* the *Irx* family genes encode transcriptional regulators that activate expression of proneural genes of the *achaete–scute* complex (AS–C) [48]. Given that the role of *Irx* transcriptional control is conserved in vertebrates [15], in which the *Irx* complex regulates proneural gene expression in the nervous system [15,49], it seemed likely that this transcriptional regulatory function would also be conserved in mosquitoes. In support of this, silencing of *Irx* transcripts (Supplementary Figure S1(A1,A2), Figure 2C, *p* < 0.001 vs. control-treated females) resulted in a significant decrease in transcript levels of the proneural gene *pdm2* in the adult brain of *A. aegypti* females (Supplementary Figure S1(B1,B2), Figure 2D, *p* < 0.001 vs. control-treated females). Combined, these results suggest that loss of *Irx* function impacts expression of critical proneural gene function in the nervous system, resulting in mosquito death.

These data indicate that *Irx*.447, an insecticide with a mode of action that differs from that of existing pesticides, could help combat insecticide resistance. These results, combined with other recent IRP ATSB studies [11,12], suggest that it may be useful to develop yeast strains that express multiple shRNAs, each targeting different genes. For example, yeast that expresses *Irx*.447 shRNA in conjunction with other newly characterized broad-based larvicidal and adulticidal shRNAs, such as dop1.462 [11] and/or Sh.463 [12] shRNAs, could be constructed. Combining two or more different shRNAs, each with a different mode of action, could be useful for managing resistance to any single shRNA [50]. Interestingly, although mixtures of some pesticides can significantly increase costs [1], expressing two or more different shRNAs in a single strain would not be expected to significantly impact the cost of yeast IRP production or application, as both insecticides would be simultaneously produced during yeast cultivation and applied together, another advantage of yeast IRP technology.

### 3.3. Irx.447 Yeast Selectively Kills Aedes, Culex, and Anopheles Mosquitoes

**Figure 3:**
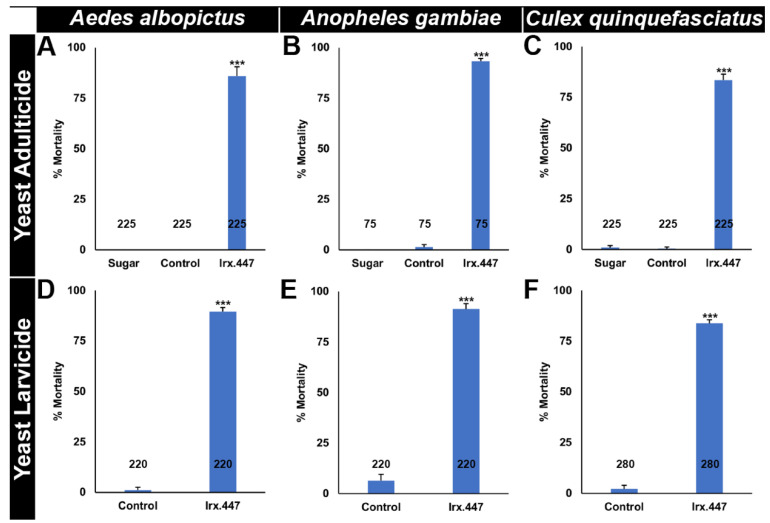
*Irx*.447 yeast is a broad-based insecticide. Oral consumption of dried inactivated *Irx*.447 yeast by larvae (**A**–**C**) or by adult females as an ATSB (**D**–**F**) induces significant mortality rates in *A. albopictus* (**A**,**C**), *A. gambiae* (**B**,**D**), and *C. quinquefasciatus* (**C**,**F**). Throughout the figure, data are shown as mean mortalities; error bars denote SEMs; *n* numbers are found below each bar in the graphs. Data were evaluated with the Student’s t-test (**A**–**C**) or with ANOVA (**D**–**F**); *** *p* < 0.001 vs. control.

An identically conserved *Irx*.447 target site is found in the sequenced genomes of many species of mosquitoes, including multiple *Anopheles* mosquitoes, *A. albopictus*, and *C. quinquefasciatus* (Table S1). It was hypothesized, on the basis of the outcomes of the ATSB and larvicide trials in *A. aegypti* (Figure 1 and Figure 2), that *Irx*.447 yeast may act as a broad-range mosquito IRP that can kill at multiple life stages in different species of mosquitoes. In support of this hypothesis, *Irx*.447 yeast ATSB was assessed in *A. gambiae* female adults, in which 100% feeding rates (Table S2) resulting in 93% ± 1% adult mortality were observed (Figure 3B, *p* < 0.001 vs. control-yeast treated adults, in which no significant death was detected). *Irx* silencing in *A. gambiae* (Supplementary Figure S2(A1–A3)) resulted in significantly reduced *pdm2* transcript levels in the adult brain (Supplementary Figure S2(B1–B3)). Consumption of *Irx*.447 yeast ATSB also induced significant mortality in *A. albopictus* (Figure 3A, *p* < 0.001 vs. control-yeast treated adults), in which 86% ± 5% adult female mortality was observed, as well as an 84% ± 3% mortality rate in *C. quinquefasciatus* (Figure 3C, *p* < 0.001 vs. control-yeast treated adults). As with *A. aegypti* and *A. gambiae,* 100% adult female feeding rates were observed in *C. quinquefasciatus,* while feeding rates in *A. albopictus* were 87 ± 1% (Table S2).

These high feeding rates (Table S2) suggest that yeast RNAi-based ATSBs may function well in the field, where many mosquito odorant attractant cues are present. The high levels of adult lethality observed in *Aedes, Anopheles,* and *Culex* mosquitoes also illustrate the promising nature of these insecticides. It will be both interesting and critical to evaluate yeast IRP-ATSB efficacy in the field, as it relates to the relative attractiveness of the baits, as well as to assess the residual activity of these pesticides upon exposure to outdoor elements. Future field trials to assess these questions are, therefore, planned, and methods for further preserving the yeast IRP activity, perhaps through formulations that enhance the stability of IRPs in a variety of different environmental conditions, both before and during use, may prove to be critical given that the ATSBs will need to be shipped, stored, and utilized in the tropics, where the formulations will need to persist through exposure to high heat. Yeast encapsulation could also enable the development of controlled and extended insecticide release formulations. Such formulations will likely be critical for the development of commercial products, which are often expected to have residual activities of several months [4].

*Irx*.447 yeast treatments also resulted in 90% ± 2% *A. albopictus* larval mortality (Figure 3D, *p* < 0.001 vs. control yeast treatment), 91% ± 3% *A. gambiae* larval mortality (Figure 3E, *p* < 0.001 vs. control yeast treatment), and 84% ± 2% *C. quinquefasciatus* larval mortality (Figure 3F; *p* < 0.001 vs. control yeast treatment). Given that larviciding is an essential component of mosquito control programs for some species of *Aedes* and *Culex* mosquitoes, the prospect of including a new class of RNAi-based larvicides to these programs is of utility and might help to address ongoing issues with resistance to existing classes of mosquito larvicides [3,51]. Although *Anopheles* mosquito control programs typically focus on adult mosquitoes, efforts to address residual transmission will need to incorporate additional mosquito control technologies [52]. The WHO [53] recommends larviciding for control when *Anopheles* breeding sites are fixed, few, and findable. Larviciding can be advantageous under certain conditions, depending on the target and the local circumstances [1]. For example, recent studies have reported that long-lasting Bti larvicides are useful for control of *A. funestus* and *A. gambiae* larvae [54,55,56,57]. *Anopheles stephensi,* a more urbanized malaria vector mosquito, can share breeding containers with *A. aegypti* [58]. It may, therefore, be possible to use the *Irx*.447 larvicide, which has a conserved target site in both mosquitoes (Table S1), to kill larvae of both species in these containers.

The ability of these insecticides to kill both larvae and adults opens opportunities to design integrated RNAi mosquito control programs in which a combination of methods, such as larvicidal treatment of breeding sites with interfering RNA larvicides, larval lethal lure-and-kill interfering RNA ovitraps [17], and RNAi-ATSBs is used simultaneously. Recent studies have uncovered a high level of acceptance of yeast RNAi-based larvicides and ovitraps among stakeholders in Trinidad and Tobago [59,60]. An engagement study in Tanzania [61] evaluated stakeholder acceptance of ivermectin-based ATSBs. The study concluded that further sensitization at the community level will be critical for educating stakeholders regarding the mode of action and use of this intervention, as most community stakeholders were not yet familiar with the ATSB paradigm. It will be interesting to gauge the acceptance of RNAi yeast-based ATSBs among stakeholders in Trinidad and elsewhere, and such studies are planned. The findings of the investigation in Tanzania [61] suggest that such studies, as well as educational campaigns that introduce stakeholders to yeast IRP ATSB technology, are essential.

Although pyrethroids, which have relatively low toxicity in humans, have been the chemicals of choice for public health control districts for several decades, widespread pyrethroid resistance threatens mosquito control strategies, necessitating the identification of novel classes of pesticides with high safety profiles [1]. In addition to evaluating the efficacy of *Irx*.447, the safety profile of this pesticide was assessed by performing toxicity screening assays in several nontarget organisms. Such assays are critical, as in silico tests are helpful but cannot exclude the possibility of off-target impacts, as one cannot predict a priori whether sites with similar, albeit not identical sequence similarity, could potentially be targeted [62]. Although *Irx*.447 yeast IRP has both mosquito larvicidal and adulticidal activities in multiple species of mosquitoes (Figure 1, Figure 2 and Figure 3), the yeast IRP did not impact survival of a group of select nontarget arthropods that were evaluated in this investigation (Table 2), including the water flea *D. magna*, the fruit fly *D. melanogaster*, the lady beetle *Hippodamia convergens,* the milkweed bug *Oncopeltus fasciatus*, and the flour beetle *T. castaneum*. These data suggest that *Irx*.447 yeast present insignificant or no threats to nontarget species, but it will of course be important to further corroborate these initial safety profile data through pursuit of expanded toxicity testing. These tests should be performed with commercial-ready yeast formulations and involve evaluations in additional species, including pollinators and vertebrate organisms, to develop a portfolio for submission to regulatory agencies.

## 4. Conclusions

Although mosquito control is a central and crucial component of mosquito-borne disease prevention strategies, insecticide resistance threatens current and future gains in the war against disease vector mosquitoes, and the identification and characterization of new active ingredients and products for mosquito control is critical [1]. The results of this investigation provide further support for the hypothesis that *Irx*.447 kills multiple species of mosquitoes at different life stages (Figure 1, Figure 2 and Figure 3) yet poses little threat to nontarget species (Table 2). These data, combined with other recent studies [5], suggest that RNAi-based yeast pesticides should be further developed as a novel class of insecticides for mosquito control. Characterization of *Irx*.447 yeast demonstrated that it functions as a dual adulticidal and larvicidal IRP with activity in *Aedes, Anopheles,* and *Culex* mosquitoes (Figure 1, Figure 2 and Figure 3), which possess a conserved *Irx* target site for this insecticide. A loss of *pdm2* transcript expression in the mosquito nervous system, which correlated with silencing of *Irx* (Figure 2D,E, Supplementary Figures S1 and S2), suggests that mortality associated with this insecticide results from disruption of proneural gene expression. Use of *Irx*.447 could facilitate the management of insecticide resistance through the addition of an insecticide with a mode of action that differs from that of existing pesticides [1].

The elimination of mosquito-borne diseases will likely require the implementation of new vector control interventions that will complement existing control measures. Thus, in addition to new insecticide classes, new paradigms will be important additions to integrated resistance management strategies [1]. To this end, the present investigation provided evidence that *Irx*.447 yeast can be successfully delivered to adult mosquitoes as an ATSB (Figure 2 and Figure 3), a sugar-baited trap, and a new paradigm for vector control [1]. These findings suggest that further development of yeast interfering RNA pesticides, the production of which is likely to be both affordable and scalable [4], should be pursued for use in ATSBs. Confirmation of *Irx*.447 yeast ATSB activity in simulated field trials performed using bait stations in cages (Figure 2 and Figure 3), as well as the analysis of *Irx*.447 yeast activity in outdoor semi-field larvicide trials (Figure 1C), indicates that these new RNAi-based technologies could potentially be useful in the field, a prospect that will be evaluated in future large-scale field trials which will be accompanied by stakeholder engagement activities and educational campaigns. Such trials will require scaled yeast production in larger fermentation-sized cultures, suggesting that the production of commercial strains that withstand fermentation, as well as the piloting and optimization of scaled yeast IRP production, would be advantageous [4].

## Figures and Tables

**Table 1:** *Irx*.447 siRNA treatments result in *Aedes aegypti* larval and adult mortality.

Trial	% Mortality	*n*	*p*-Value
**Larval soaking**			
Control siRNA	5 ± 5 *	40	<0.001
*lrx* siRNA	72.5 ± 2.5	40	
**Adult microinjection**			
Control siRNA	5	20	0.0092
*lrx* siRNA	40	20	
**ATSB feeding**			
Control siRNA	8 ± 5	37	<0.001
*lrx* siRNA	75 ± 3	42	

* Mortality rates and standard errors of the mean (SEM), *n* numbers, and the *p*-value found in Fischer’s exact tests comparing *Irx*.447 siRNA-treated vs. control-treated *A. aegypti* are shown.

**Table 2:** Viability of nontarget arthropods following consumption of *Irx*.447 yeast.

		% Survival	
Test organism	*n*/ Treatment	Control Yeast	*lrx*.447 Yeast
*D. melanogaster* larvae	60 *	100 ± 0	100 ± 0
*D. melanogaster* adults	60	100 ± 0	100 ± 0
*Tribolium* adults	40	100 ± 0	100 ± 0
*Oncopeltus fasciatus* adults	20	80 ± 7	90 ± 14
*Hippodamia convergens* adults	20	100 ± 0	100 ± 0
*Daphnia magna* adults	40	100 ± 0	100 ± 0

* Survival was assessed after consumption of *Irx*.447 yeast or control interfering RNA yeast delivered as an ATSB to the indicated arthropods. Mean percentages of survival with SEMs, as well as *n* numbers corresponding to the number of animals treated, are indicated. Fisher’s exact test comparisons did not reveal any significant differences in survival between insecticide-treated and control interfering RNA-treated arthropods.

## Data Availability

All data are provided within the text and supplementary materials of this manuscript.

## Supplementary Materials

The following are available online at https://www.mdpi.com/article/10.3390/insects12110986/s1: Table S1. The *Irx*.447 target site conserved in mosquitoes was not found in non-mosquito genomes [47]; Table S2. Mosquito ATSB feeding rates observed in laboratory simulated field trials; Figure S1. *Irx*.447 yeast ATSB induces target gene silencing and reduces *pdm2* expression in *A. aegypti;* Figure S2. *Irx*.447 yeast ATSB induces silencing of the *Irx* target gene and significantly reduces *pdm2* expression in *A. gambiae.*
