# Supplementary material for: A Yeast RNA-Interference Pesticide Targeting the Irx Gene Functions as a Broad-Based Mosquito Larvicide and Adulticide
Source: Insects. 2021 Nov 2;12(11):986. doi: 10.3390/insects12110986 (PMC8622680; doi:10.3390/insects12110986)
Supplement: Supplementary file 1 [file insects-12-00986-s001.zip › insects-1438033-supplementary.pdf]

insects

MDPI

Supplementary Material

Table S1. The *Irx*.447 target site conserved in mosquitoes was not found in non-mosquito genomes.

Species/Taxon Group	Gene/Contig
*Aedes aegypti**	AAEL007505
Aedes albopictus	AALF026228
Anopheles albimanus	AALB016292
Anopheles arabiensis	AARA016724
Anopheles atroparvus	AatrE3_2L
Anopheles coluzzii	ACON005010
Anopheles culicafacies	KI423683
Anopheles darlingi	ADMH02001638
Anopheles dirus	ADIR015668
Anopheles epiroticus	KB672242
Anopheles farauti	KI915044
Anopheles funestus	AFUN016038
Anopheles gambiae	AGAP005010
Anopheles maculatus	AMAM012126
Anopheles melas	AXCO02001637
Anopheles merus	AMEM019330
Anopheles minimus	AMIN011495
Anopheles quadriannulatus	AQUA016748
Anopheles sinensis	KE525340
Anopheles stephensi	ASTEI09300
Culex pipiens pallens	LOC120426920
Culex quinquefasciatus	CPIJ000125
Amphibians	None
Arthropods (non-mosquito)	None
Birds	None
Fish	None
Fungi	None
Human	None
Mammals	None
Plants	None
Reptiles	None

* The *Irx*.447 target site is identically conserved in multiple disease vector mosquito *Irx* genes, but this identical 21 bp target site was not detected in the genomes of non-target species in the National Center Biotechnology Information (NCBI) database [47].

Table S2. Mosquito ATSB feeding rates observed in laboratory simulated field trials.

	Species	Feeding rate (%)		n/treatment
		Control	lrx.447	
siRNA/ATSB	*Aedes aegypti**	57 + 7	63 + 12	68
yeast/ATSB	*Aedes aegypti*	100 ± 0	100 ± 0	150
	Anopheles gambiae	100 ± 0	100 ± 0	75
	Culex quinquefasciatus	100 ± 0	100 ± 0	225
	Aedes albopictus	87 ± 0.5	87 ± 2	225

* The percentages of total insects that took sugar meals including each of the indicated treatments are shown. The species of mosquito, feeding rates with SEMs, as well as the n number of individuals receiving each treatment are shown. No significant differences

between feeding rates for the insecticidal or control treatments were identified (Student's t-test).

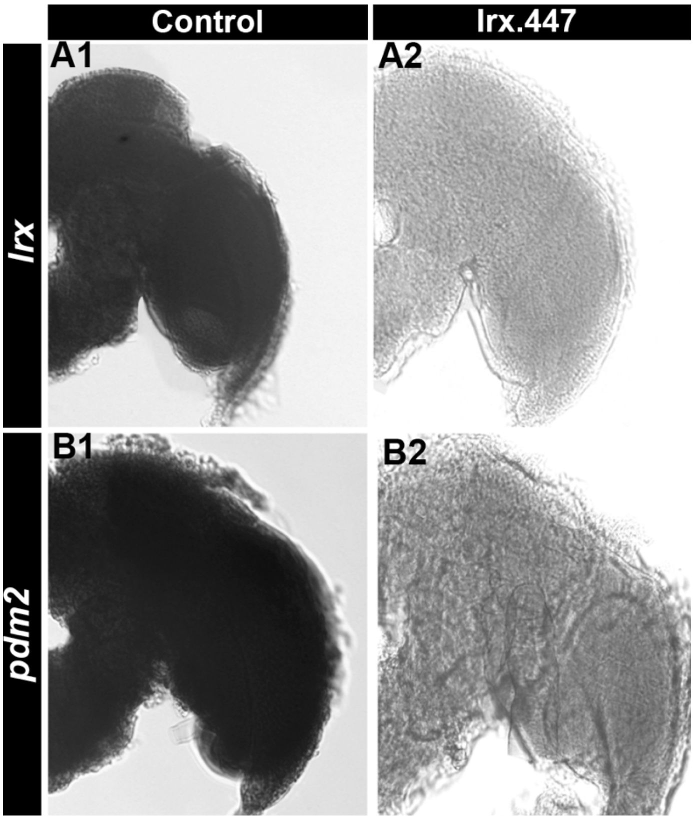

Figure S1. *Irx*.447 yeast ATSB induces target gene silencing and decreased *pdm2* expression in *A. aegypti*. Consumption of *Irx*.447 yeast ATSB (**A2**,**B2**) resulted in a significant loss of *Irx* (**A2**) and *pdm2* (**B2**) transcripts in the adult female *A. aegypti* brain (compare to control-treated female brains in **A1** and **B1**).

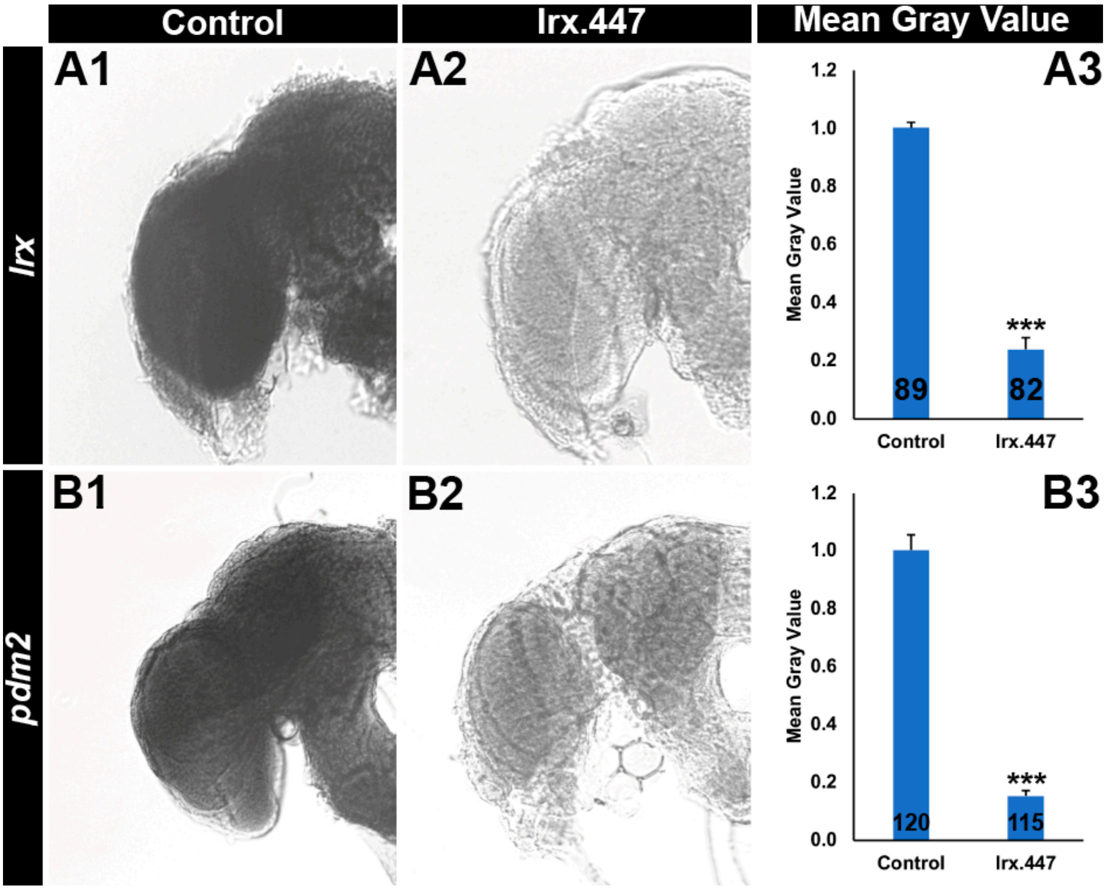

Figure S2. *Irx*.447 yeast ATSB results in target gene silencing and significantly impacts *pdm2* expression in *A. gambiae*. Consumption of *Irx*.447 yeast ATSB resulted in a significant reduction in *Irx* (**A2**,**A3**) and *pdm2* (**B2**,**B3**) transcripts in the adult female *A. gambiae* brain (compare to control-treated female brains in **A1** and **B1**), as evidenced by mean gray value analyses (**A3**,**B3**). The numbers of brains analyzed (*n*) are specified below each bar in the graphs; *** = $p < 0.001$ vs. control (Student's t-test).

References

47. Sayers, E.W.; Beck, J.; Bolton, E.E.; Bourexis, D.; Brister, J.R.; Canese, K.; Comeau, D.C.; Funk, K.; Kim, S.; Klimke, W.; et al. Database resources of the national center for biotechnology information. *Nucleic Acids Res.* **2021**, *49*, D10–D17. https://doi.org/10.1093/nar/gkaa892.
